# Supplementary material for: Looming Angry Faces: Preliminary Evidence of Differential Electrophysiological Dynamics for Filtered Stimuli via Low and High Spatial Frequencies
Source: Brain Sci. 2024 Jan 19;14(1):98. doi: 10.3390/brainsci14010098 (PMC10813450; doi:10.3390/brainsci14010098)
Supplement: Supplementary file 1 [file brainsci-14-00098-s001.zip › brainsci-2704984-supplementary.pdf]

Supplementary Materials

1. Tables of the Descriptive Statistics of the Reported ERPs

Experiment 1

Table S1. Means and Standard Deviations (SD) of the P1 amplitudes (μV) for each Motion Direction x Emotion condition at the HSF level *(AP: approaching; RE: receding; STAT: static; ANG: angry; NEU: neutral;).*

Motion	EMO	Mean	SD
AP	ANG	2.926	2.053
	NEU	2.868	1.980
RE	ANG	2.855	2.367
	NEU	2.857	2.163
STAT	ANG	2.909	1.965
	NEU	2.760	2.037

Table S2. Means and Standard Deviations (SD) of the P1 amplitudes (μV) for each Motion Direction x Emotion condition at the LSF level *(AP: approaching; RE: receding; STAT: static; ANG: angry; NEU: neutral;).*

Motion	EMO	Mean	SD
AP	ANG	3.265	1.856
	NEU	3.310	1.677
RE	ANG	3.452	1.541
	NEU	3.200	1.505
STAT	ANG	3.263	1.341
	NEU	3.330	1.521

Table S3. Means and Standard Deviations (SD) of the N170 amplitudes (μV) for each ROI x Motion Direction x Emotion condition at the HSF level *(L: left; R: right; AP: approaching; RE: receding; STAT: static; ANG: angry; NEU: neutral;).*

ROI	MOTION	EMOTION	Mean	SD
L	AP	ANG	-4.391	3.705
		NEU	-4.188	3.805
	RE	ANG	-4.061	3.355

ROI	MOTION	EMOTION	Mean	SD
		NEU	-4.310	3.678
	STAT	ANG	-3.959	3.415
		NEU	-3.775	3.734
R	AP	ANG	-3.639	3.219
		NEU	-3.869	3.078
	RE	ANG	-4.083	3.187
		NEU	-4.136	3.116
	STAT	ANG	-3.365	3.016
		NEU	-3.279	2.898

Table S4. Means and Standard Deviations (SD) of the N170 amplitudes (μV) for each ROI x Motion Direction x Emotion condition at the LSF level *(L: left; R: right; AP: approaching; RE: receding; STAT: static; ANG: angry; NEU: neutral;).*

ROI	MOTION	EMOTION	Mean	SD
L	AP	ANG	-3.623	2.786
		NEU	-3.786	3.000
	RE	ANG	-3.452	2.745
		NEU	-3.408	2.681
	STAT	ANG	-3.368	2.608
		NEU	-3.444	2.808
R	AP	ANG	-3.835	2.993
		NEU	-3.645	3.167
	RE	ANG	-3.484	2.833
		NEU	-3.183	3.225
	STAT	ANG	-3.585	3.031
		NEU	-3.280	2.950

Table S5. Means and Standard Deviations (SD) of the P2 amplitudes (μV) for each Motion Direction x Emotion condition at the HSF level *(AP: approaching; RE: receding; STAT: static; ANG: angry; NEU: neutral;).*

MOTION	EMOTION	Mean	SD
AP	ANG	4.132	3.151
	NEU	3.952	3.081
RE	ANG	4.605	3.306
	NEU	4.600	3.192
STAT	ANG	5.037	3.480
	NEU	4.861	3.365

Table S6. Means and Standard Deviations (SD) of the P2 amplitudes (μV) for each Motion Direction x Emotion condition at the LSF level *(AP: approaching; RE: receding; STAT: static; ANG: angry; NEU: neutral;).*

MOTION	EMOTION	Mean	SD
AP	ANG	4.825	2.278
	NEU	4.564	1.825
RE	ANG	5.459	2.289
	NEU	5.354	2.096
STAT	ANG	5.294	2.090
	NEU	5.359	2.023

Experiment 2

Table S7. Means and Standard Deviations (SD) of the P1 amplitudes (μV) for each Motion Direction x Emotion condition at the unfiltered/BSF, HSF, and LSF level, respectively *(AP: approaching; RE: receding; ANG: angry; NEU: neutral;).*

Spatial Frequency	Motion	Emotion	Mean	SD
BSF	AP	ANG	3.751	2.394
		NEU	3.471	2.455
	RE	ANG	4.141	2.401
		NEU	3.736	2.393
HSF	AP	ANG	3.269	2.302
		NEU	3.152	2.272
	RE	ANG	3.108	2.269
		NEU	3.230	2.122
LSF	AP	ANG	3.908	2.614
		NEU	3.613	2.215
	RE	ANG	4.071	2.731
		NEU	3.955	2.424

Table S8. Means and Standard Deviations (SD) of the N170 amplitudes (μV) for each ROI x Motion Direction x Emotion condition at the unfiltered/BSF level *(L: left; R: right; AP: approaching; RE: receding; ANG: angry; NEU: neutral;).*

ROI	MOTION	EMOTION	Mean	SD
L	AP	ANG	-4.037	2.494
		NEU	-3.973	2.331
	RE	ANG	-4.175	2.399
		NEU	-4.034	2.362
R	AP	ANG	-4.445	3.052
		NEU	-3.943	2.966
	RE	ANG	-4.658	3.152
		NEU	-4.250	2.958

Table S9. Means and Standard Deviations (SD) of the N170 amplitudes (μV) for each ROI x Motion Direction x Emotion condition at the HSF level *(L: left; R: right; AP: approaching; RE: receding; ANG: angry; NEU: neutral;).*

ROI	MOTION	EMOTION	Mean	SD
L	AP	ANG	-3.663	2.516

ROI	MOTION	EMOTION	Mean	SD
		NEU	-3.938	2.755
	RE	ANG	-4.026	2.623
		NEU	-4.285	2.570
R	AP	ANG	-4.040	3.858
		NEU	-4.089	3.792
	RE	ANG	-4.166	3.998
		NEU	-4.276	3.712

Table S10. Means and Standard Deviations (SD) of the N170 amplitudes (μV) for each ROI x Motion Direction x Emotion condition at the LSF level *(L: left; R: right; AP: approaching; RE: receding; ANG: angry; NEU: neutral;).*

ROI	MOTION	EMOTION	Mean	SD
L	AP	ANG	-3.775	2.390
		NEU	-3.691	2.281
	RE	ANG	-3.830	2.413
		NEU	-3.467	2.457
R	AP	ANG	-3.823	2.738
		NEU	-3.770	2.699
	RE	ANG	-4.048	2.914
		NEU	-3.986	3.158

Table S11. Means and Standard Deviations (SD) of the P2 amplitudes (μV) for each Motion Direction x Emotion condition at the BSF, HSF, and LSF level, respectively *(AP: approaching; RE: receding; ANG: angry; NEU: neutral;).*

		BSF		HSF		LSF	
**Motion**	**Emotion**	**Mean**	**SD**	**Mean**	**SD**	**Mean**	**SD**
AP	ANG	3.658	3.359	0.379	3.374	4.222	3.625
	NEU	3.960	3.597	0.553	3.549	4.270	3.792
RE	ANG	4.257	3.097	1.044	3.184	5.027	3.797
	NEU	4.215	3.213	1.417	3.134	5.182	3.554

2. Grand Traces of Each Condition of the Reported ERPs

Experiment 1

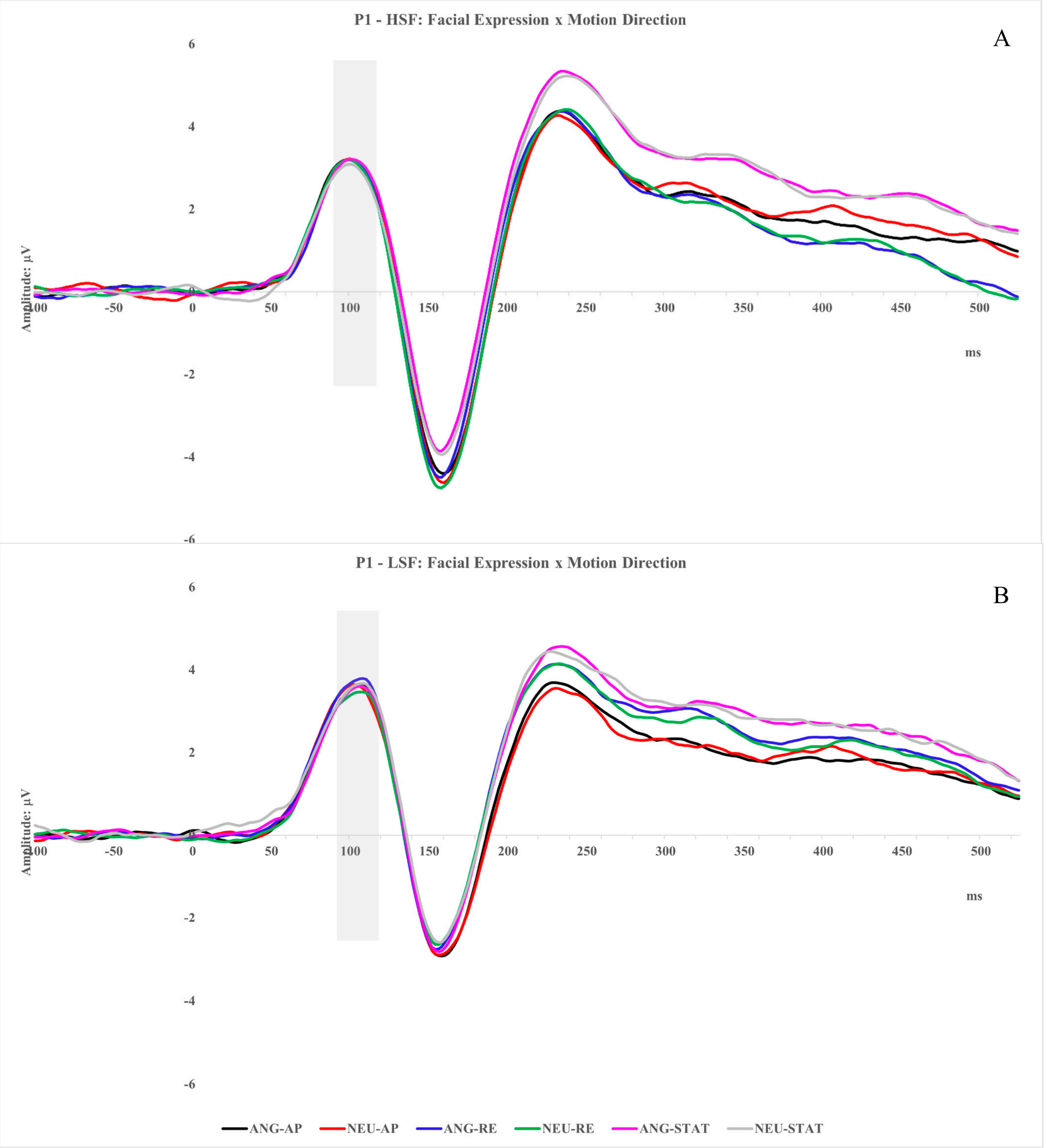

Figure S1. Grand ERP traces for each condition of the P1 at HSF level (A) and LSF level (B), respectively. *AP: approaching; RE: receding; ST: static; ANG: angry; NEU: neutral.*

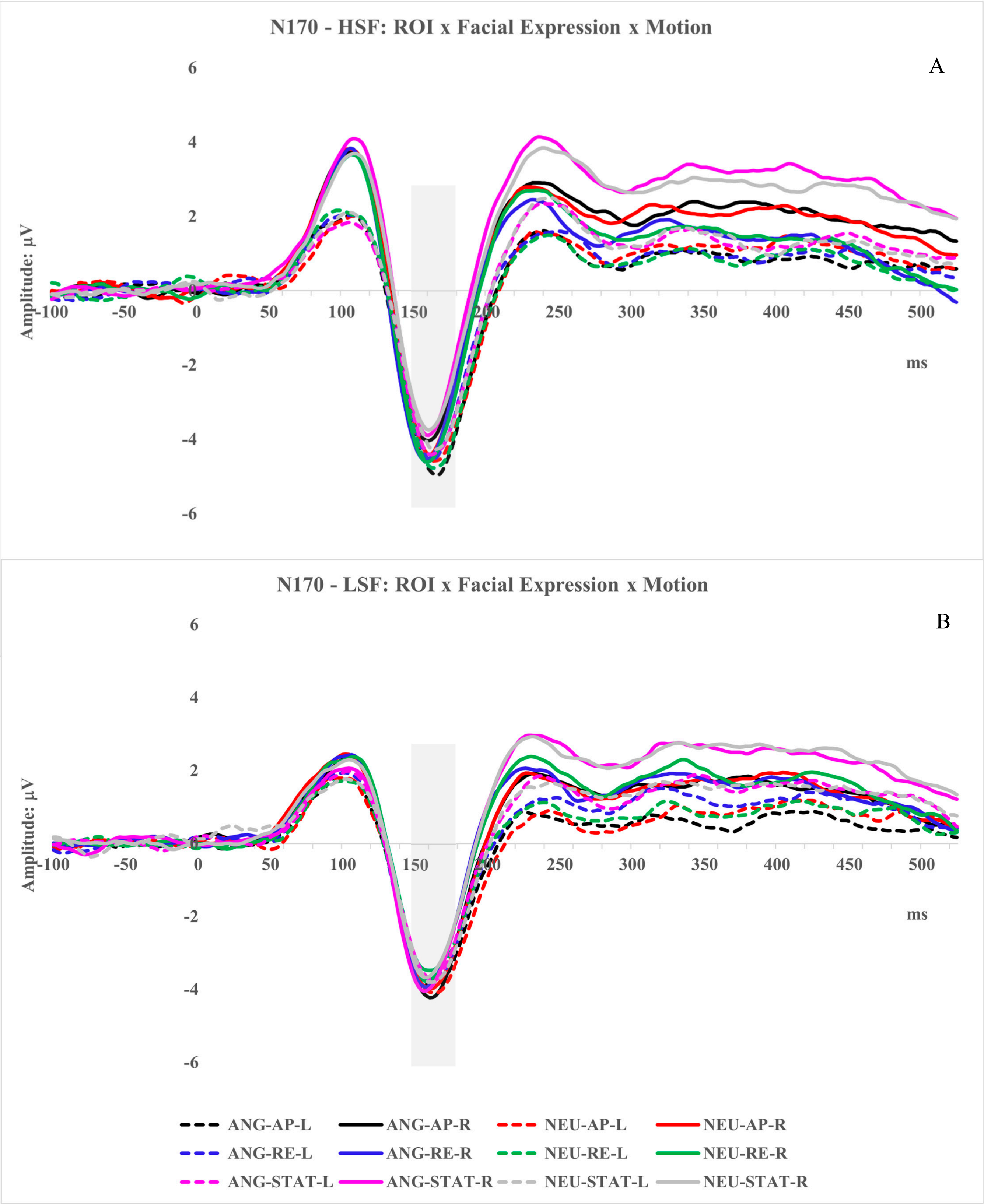

Figure S2. Grand ERP traces for each condition of the N170 at HSF level (A) and LSF level (B), respectively. *L: left ROI; R: right ROI; AP: approaching; RE: receding; ST: static; ANG: angry; NEU: neutral.*

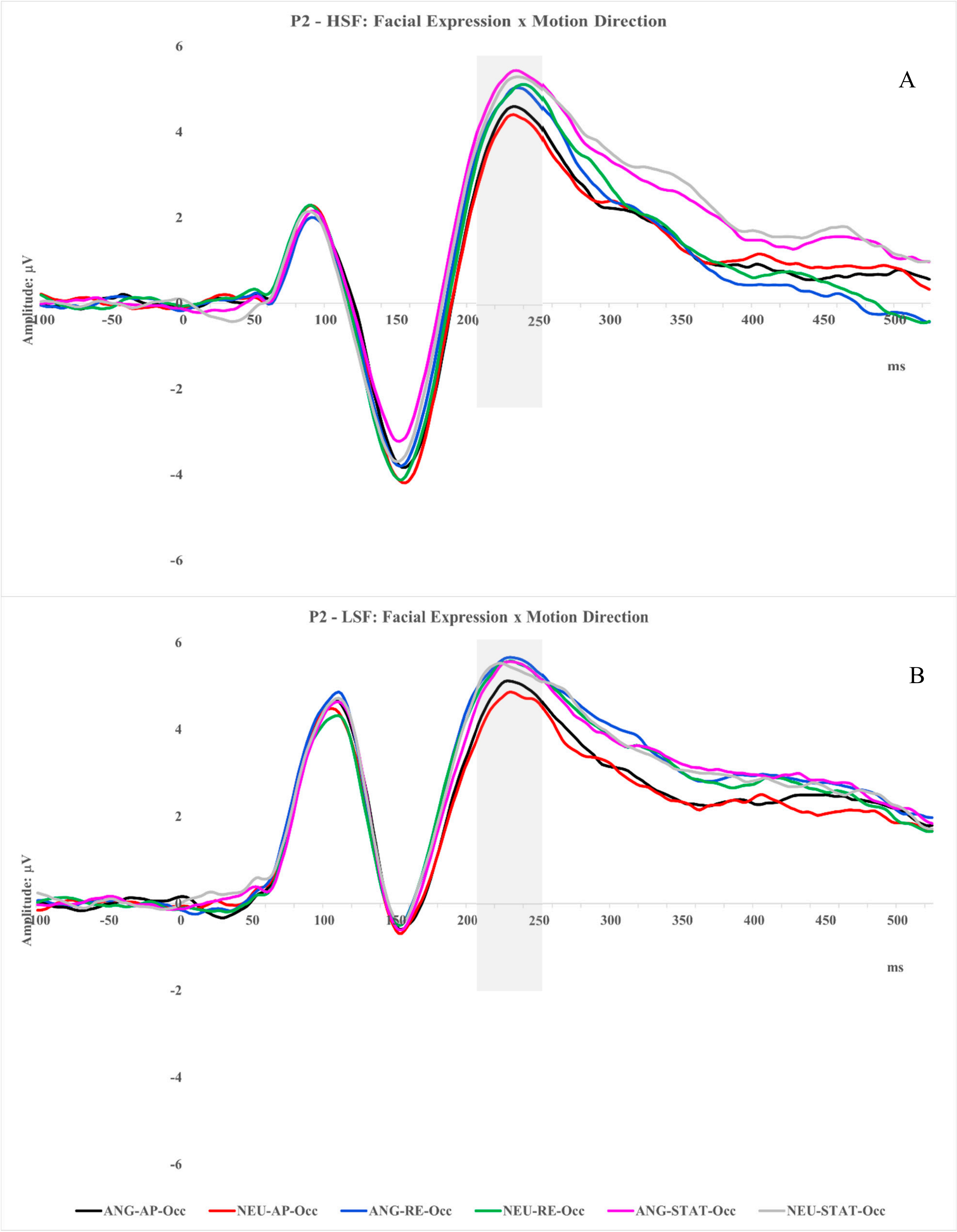

Figure S3. Grand ERP traces for each condition of the P2 at HSF (A) and LSF (B) level. *AP: approaching; RE: receding; ST: static; ANG: angry; NEU: neutral.*

Experiment 2

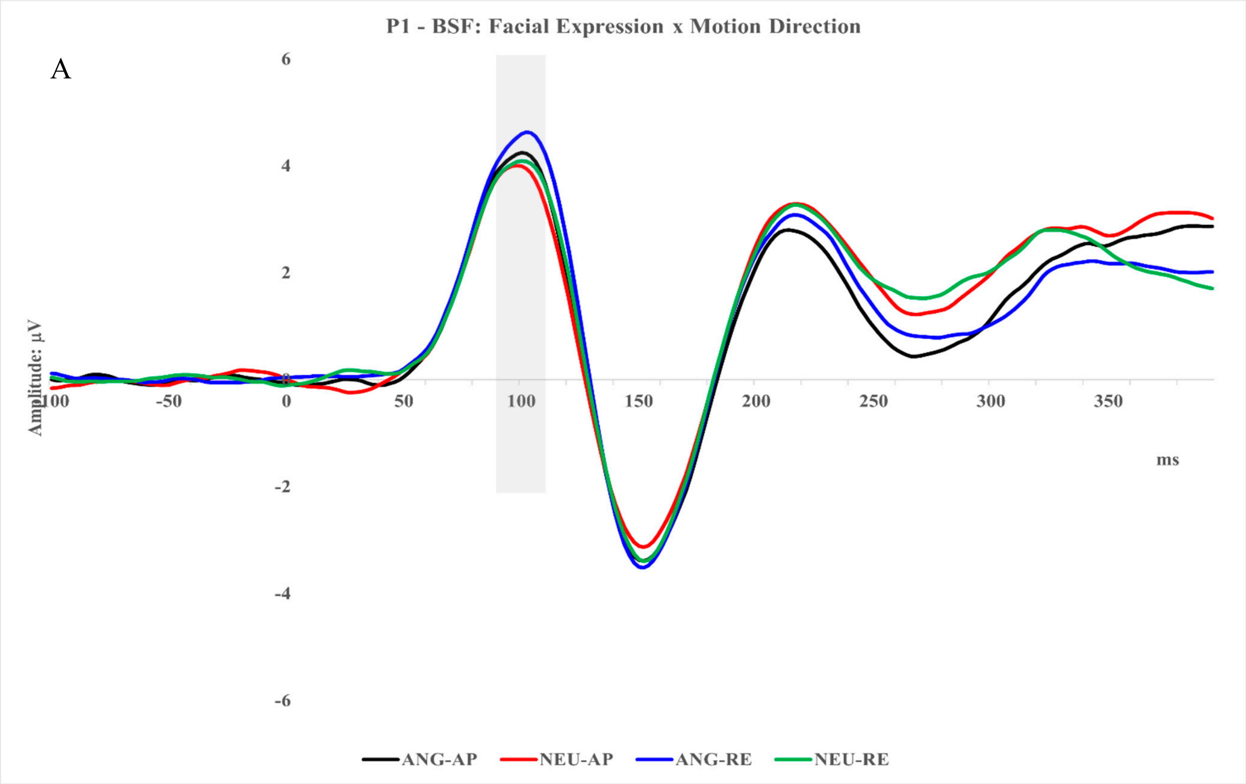

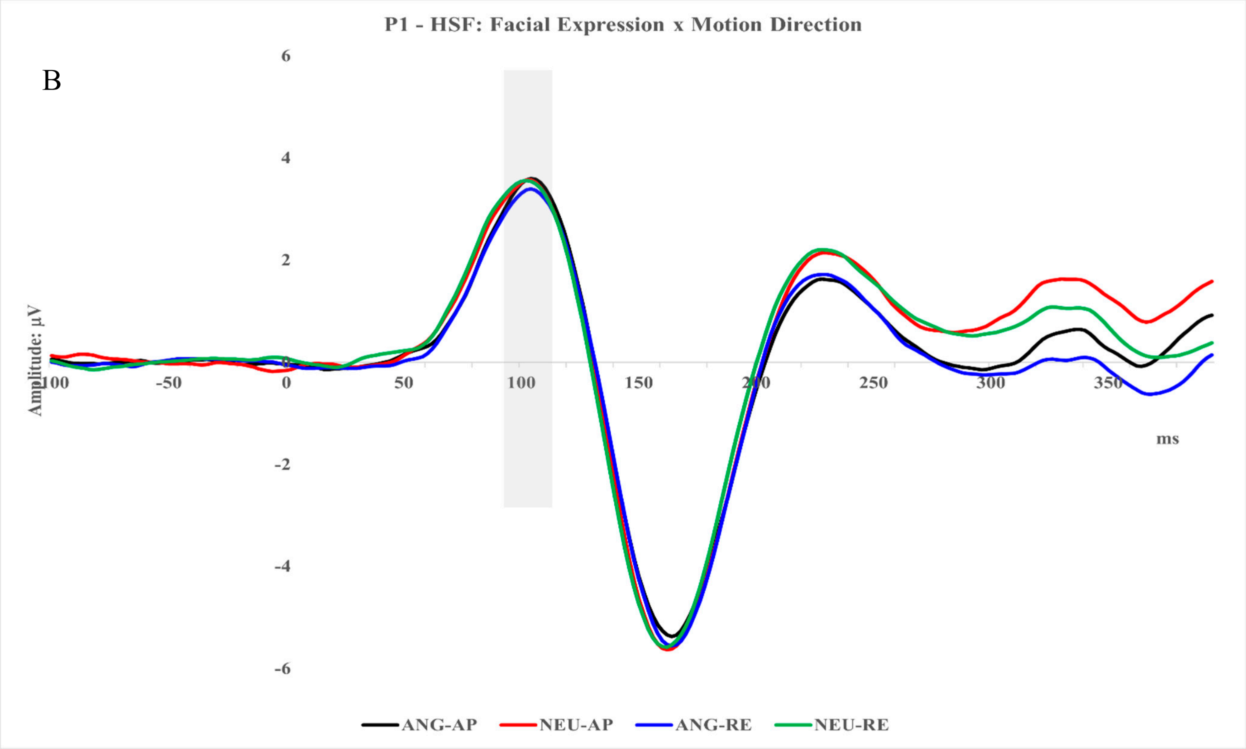

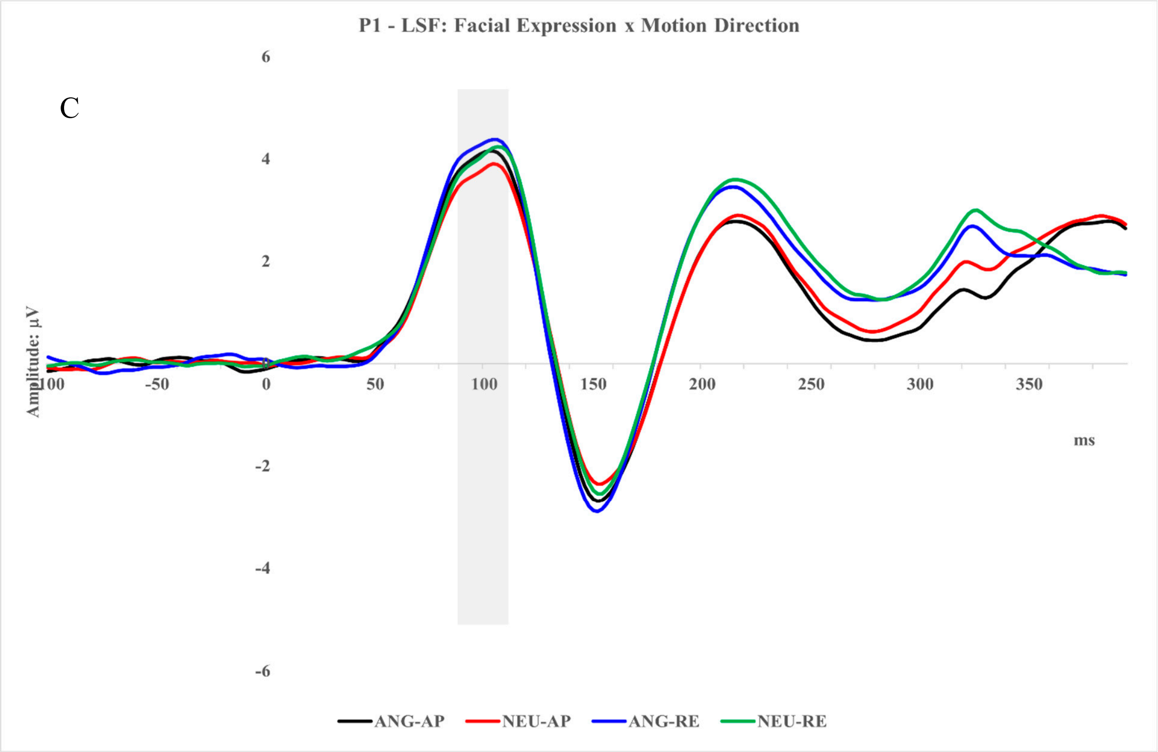

Figure S4. Grand ERP traces for each condition of the P1 at BSF (A), HSF level (B) and LSF level (C), respectively. *AP: approaching; RE: receding; ANG: angry; NEU: neutral.*

A

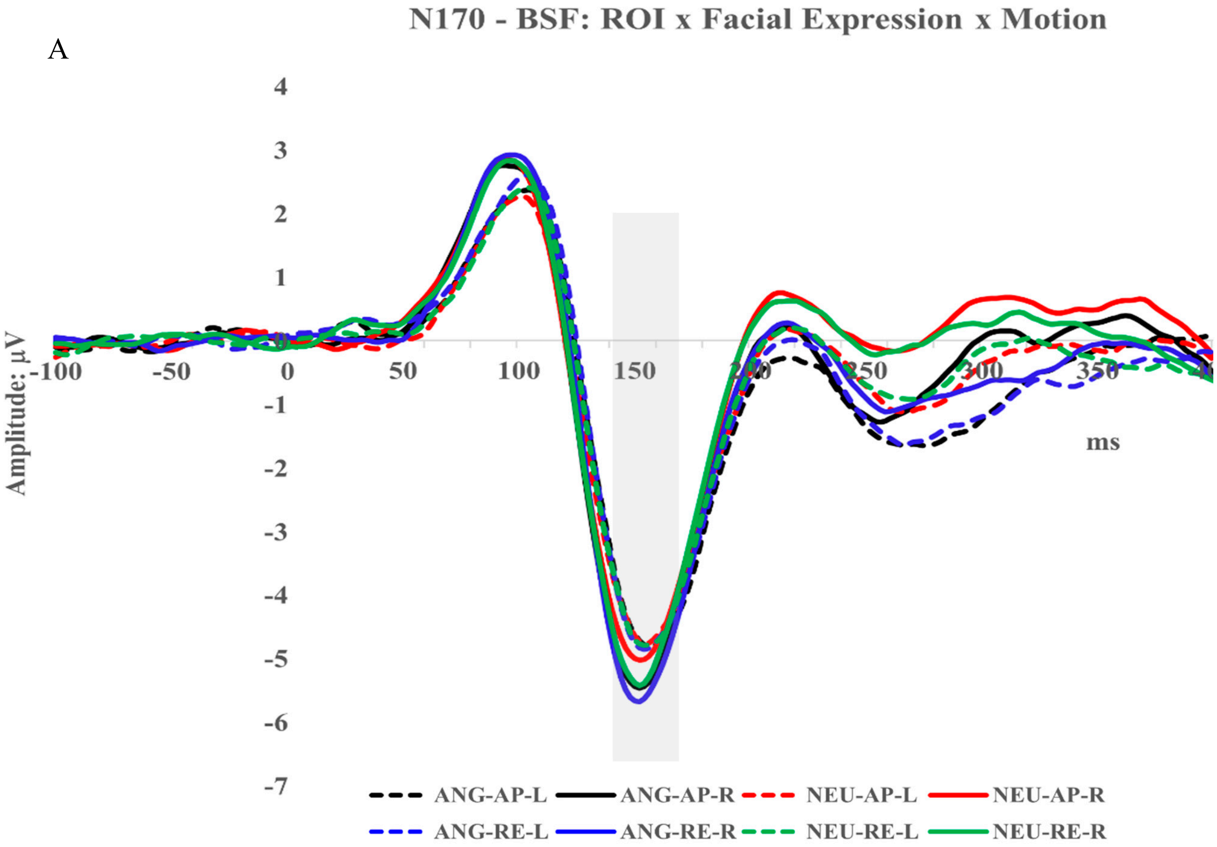
N170 - BSF: ROI x Facial Expression x Motion
Amplitude: µV
ms
ANG-AP-L
ANG-AP-R
NEU-AP-L
NEU-AP-R
ANG-RE-L
ANG-RE-R
NEU-RE-L
NEU-RE-R

B

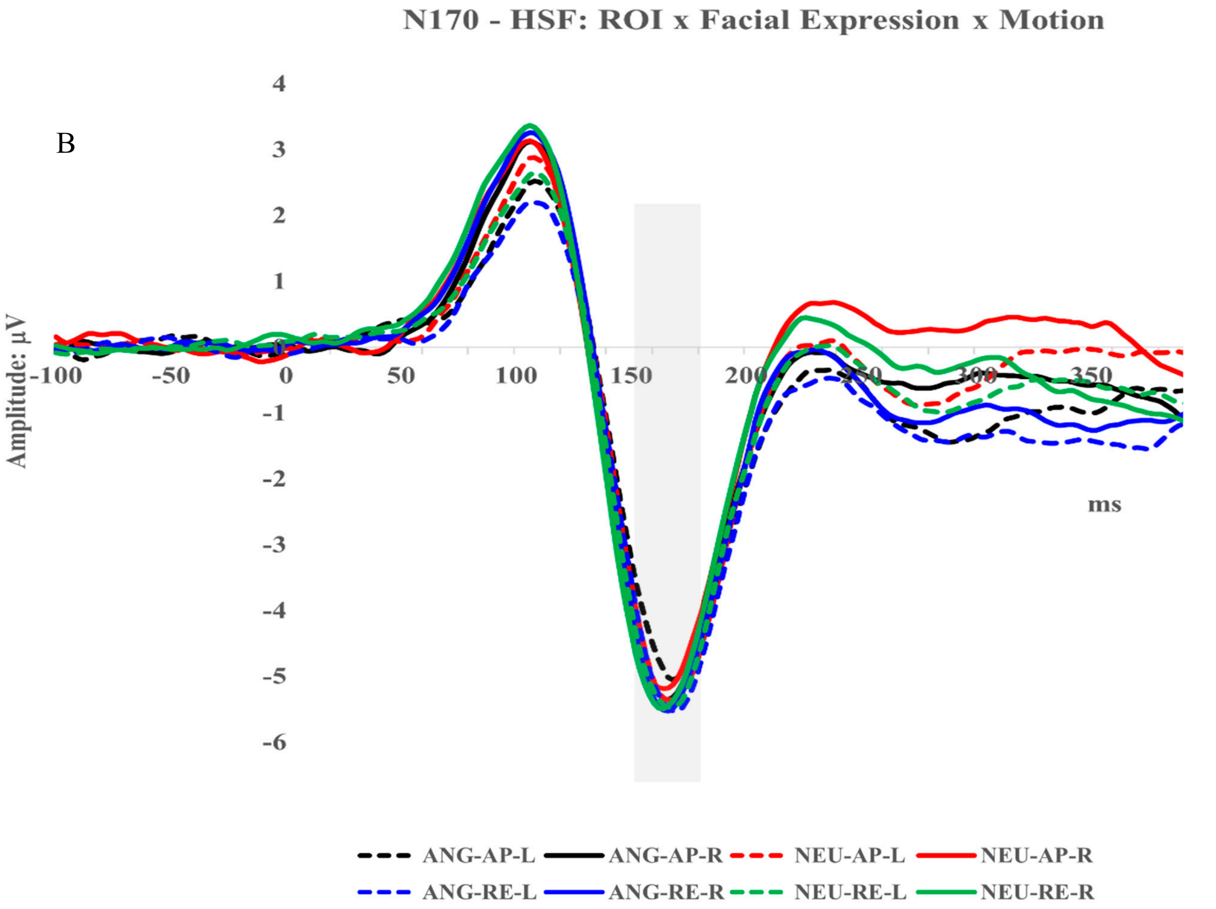
N170 - HSF: ROI x Facial Expression x Motion
Amplitude: µV
ms
ANG-AP-L
ANG-AP-R
NEU-AP-L
NEU-AP-R
ANG-RE-L
ANG-RE-R
NEU-RE-L
NEU-RE-R

C

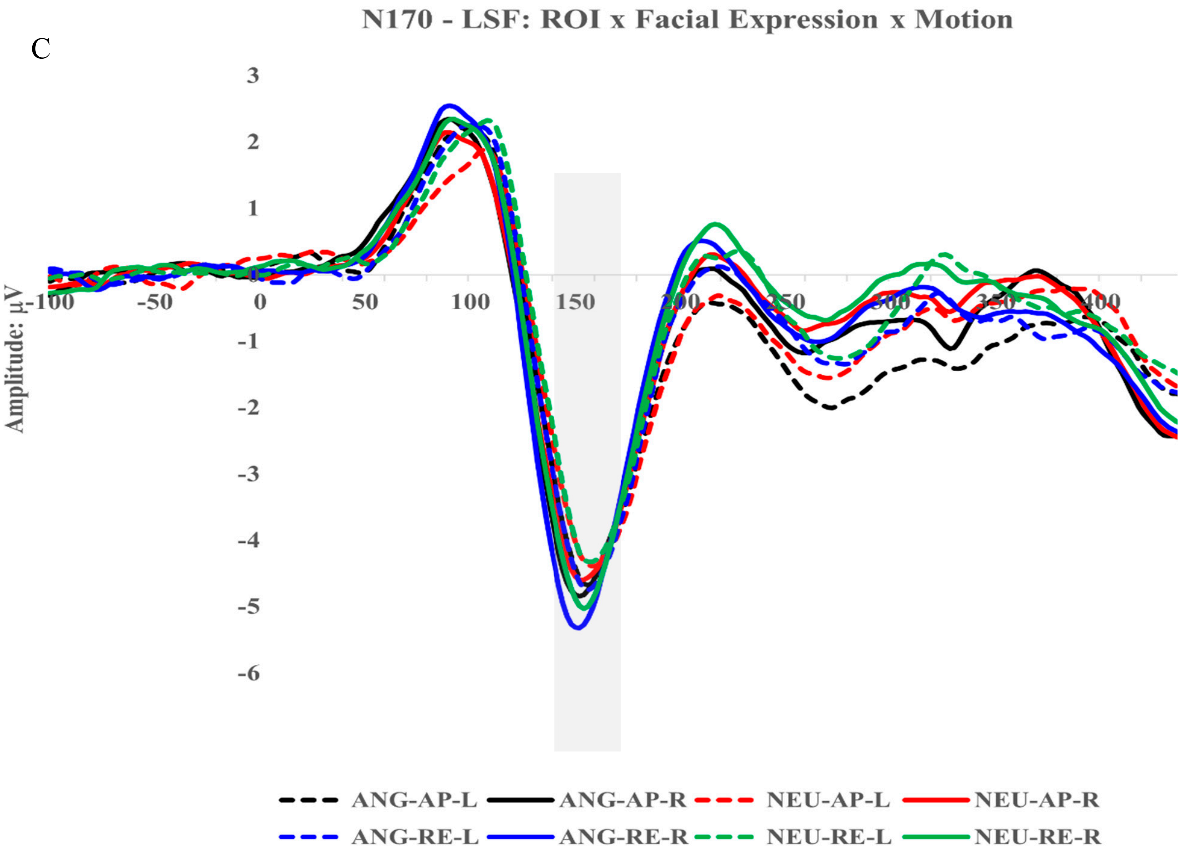

Figure S5. Grand ERP traces for each condition of the N170 at BSF (A), HSF level (B) and LSF level (C), respectively. *AP: approaching; RE: receding; ANG: angry; NEU: neutral; L:left; R:right..*

A

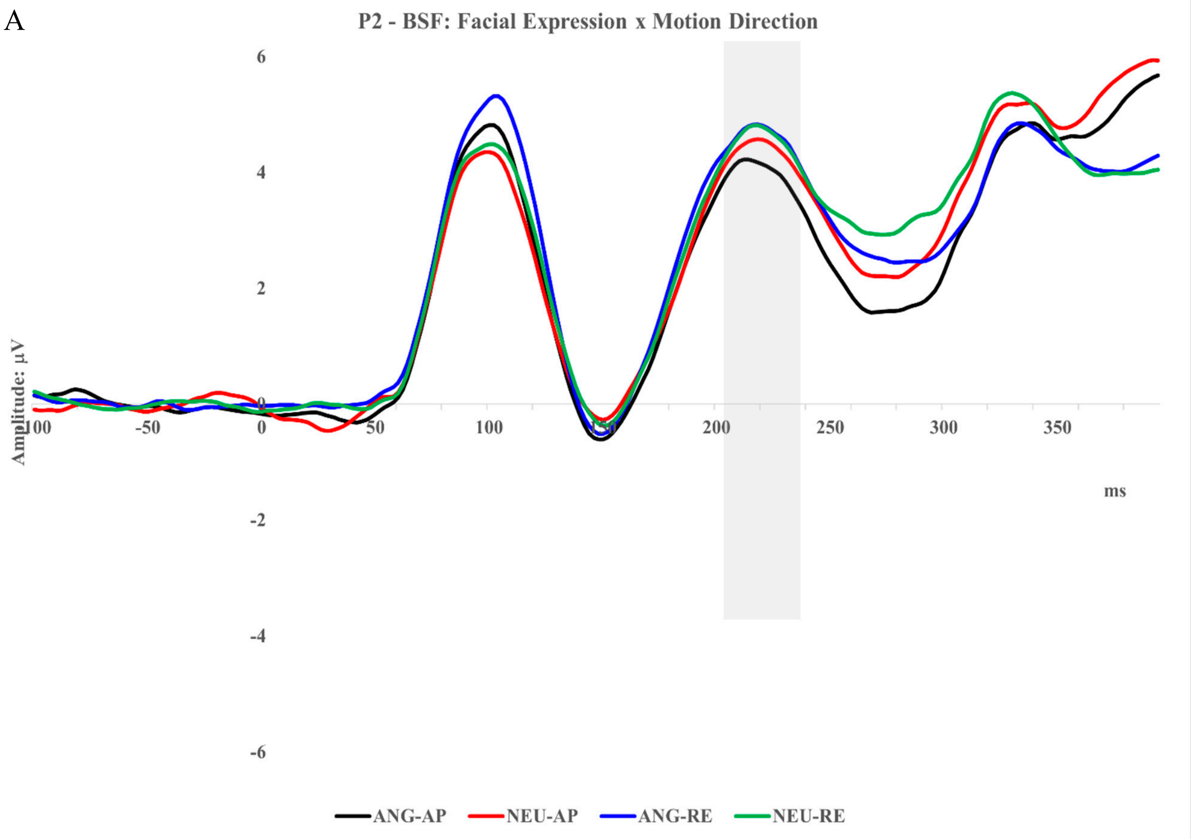
P2 - BSF: Facial Expression x Motion Direction
6
4
2
0
-2
-4
-6
Amplitude: µV
-100
-50
0
50
100
150
200
250
300
350
ms
ANG-AP
NEU-AP
ANG-RE
NEU-RE

B

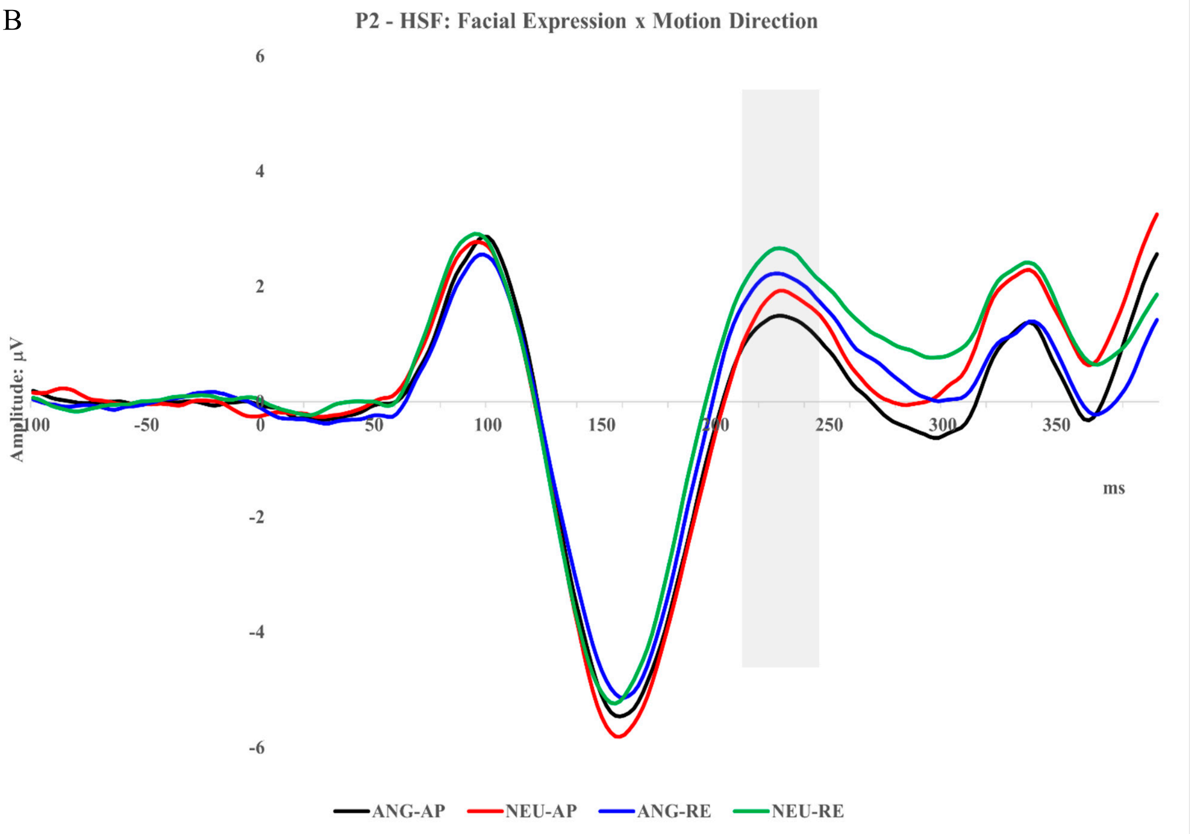
P2 - HSF: Facial Expression x Motion Direction
6
4
2
0
-2
-4
-6
Amplitude: µV
-100
-50
0
50
100
150
200
250
300
350
ms
ANG-AP
NEU-AP
ANG-RE
NEU-RE

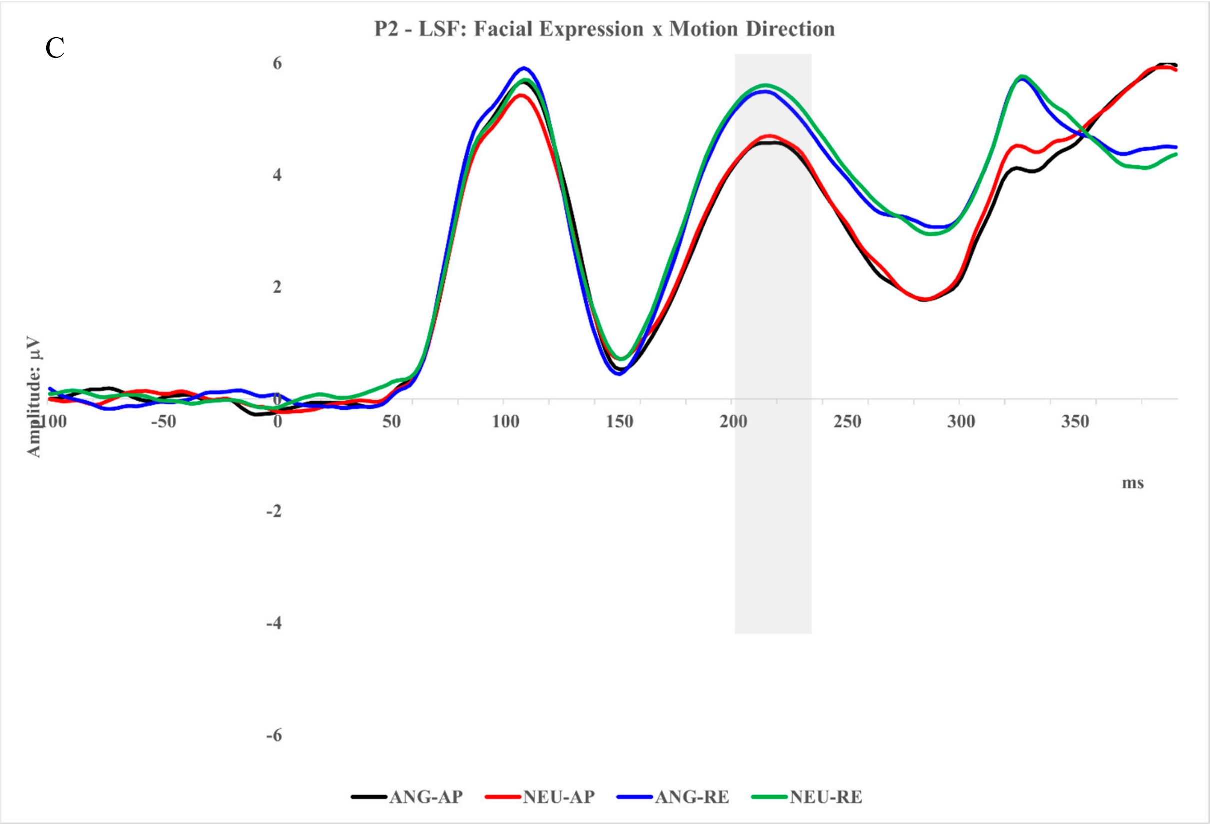

Figure S6. Grand ERP traces for each condition of the P2 at BSF (A), HSF level (B) and LSF level (C), respectively. *AP: approaching; RE: receding; ANG: angry; NEU: neutral.*
